# Synchronous Fibromatosis Indistinguishable from Suspected Synchronous Gastrointestinal Stromal Tumor: A Case Report

**DOI:** 10.31729/jnma.6516

**Published:** 2021-09-30

**Authors:** Anup Chalise, Ashish Prasad Rajbhandari, Ramesh Dhakhwa

**Affiliations:** 1 Department of Surgery, Nepal Medical College and Teaching Hospital, Attarkhel, Kathmandu, Nepal; 2 Department of Pathology, Kathmandu Medical College and Teaching Hospital, Sinamangal, Kathmandu, Nepal

**Keywords:** *case report*, *desmoid*, *fibromatosis*, *immunohistochemistry*

## Abstract

Desmoid tumors most commonly occur in the anterior abdominal wall in approximately 50% of cases and are locally aggressive. We describe a case of a 38-year-old lady who was investigated as a case of gastrointestinal tumor. Post-operative immunohistochemistry staining showed the presence of a synchronous desmoid in the abdominal wall and proximal ileum. Wide local excision remains the gold-standard of treatment with pharmacotherapeutics and radiotherapy serving as adjuvant or palliative treatment options.

## INTRODUCTION

Desmoid tumors, although not malignant, are associated in females, especially during and after pregnancy. These most commonly occur in the anterior abdominal wall in approximately 50% of cases.^1^ However, they are locally aggressive.^2^ These usually occur postoperatively but may arise in the pre-operative setting.^1^ Desmoid tumors can occur in the mesentery of the intestine, when associated with familial adenomatous polyposis (FAP).^3^

## CASE REPORT

Our report describes a 38-year-old male who presented to us with an abdominal lump, noticed 6 months prior. The lump was gradually increasing in size and was more prominent during exercise. He also noted early satiety since the last 2 months, but denied weight loss, nausea or vomiting. The history was also insignificant for hematemesis, hematochezia or melena. On examination, the patient wasn't pale, icteric, edematous or dehydrated. No cervical lymph nodes were palpable. Abdomen was soft, without tenderness or visceromegaly. There was an approximately 6x8cm firm, fluctuant, fixed mass in the mid upper abdomen, without any skin changes suggestive of inflammation, which became more prominent on raising the legs.

**Figure 1:**
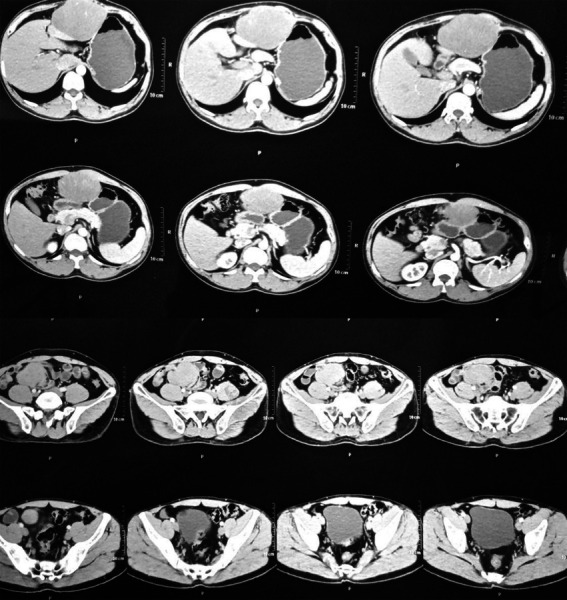
CT scan showing the described findings.

The patient underwent an ultrasonogram, which revealed a well-defined hypoechoic lesion about 8x 5cm in the subhepatic and 6 x 5cm lesion in the right pelvic lesion with minimal vascularity and minimal ascites. The patient further underwent a CT scan of the abdomen, which showed two well defined soft tissue lesions within the abdominal and pelvic cavity (Figure 1).

The abdominal mass was well defined, oval, ~76 x 109mm showing heterogeneous post contrast enhancement, without calcification or cavitation. The mass was noted to lie in between the greater curvature of stomach and liver with loss of fat plane with anterior abdominal wall, stomach and liver. Another lesion in the pelvic cavity was ~59 x 67mm on the right side at the level of the pelvic brim with similar findings. Differential diagnoses provided were gastrointestinal stromal tumor, nerve sheath tumor, soft tissue sarcoma and desmoid tumor.

**Figure 2:**
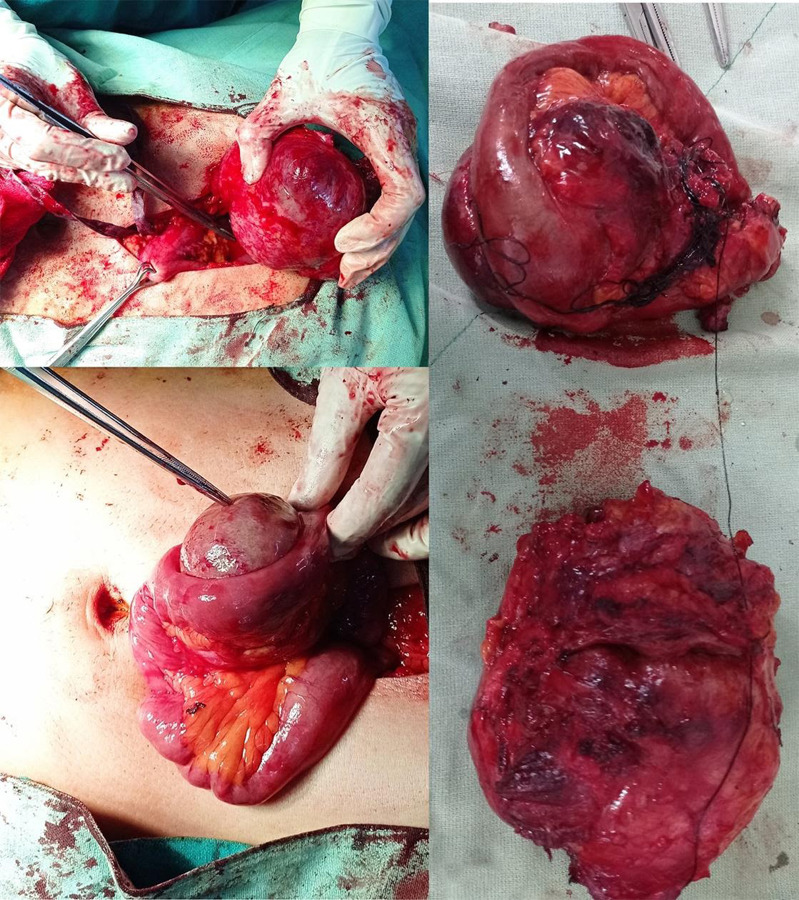
Intraoperative and postoperative pictures showing the resected specimen.

With normal preoperative investigation, the patient underwent exploratory laparotomy, resection of the lumps and primary anastomosis (Figure 2).

**Figure 3:**
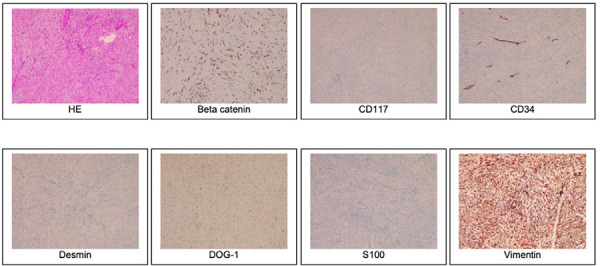
Immunohistochemistry reports showing features consistent with desmoid tumor.

Intra-op findings revealed a 7 x 8 x 4cm lump attached to the muscular layer of the abdominal wall, extending up to the peritoneal layer, without any intraperitoneal extension. Another lump 10 x 8 x 6cm was noted to be arising from the proximal ileum, which was mobile and not invading the serosa of the small bowel. Post-op immunohistochemistry was reactive for SMA, b-catenin and vimentin, consistent with features of desmoid tumor (Figure 3).

One month post-operatively the patient underwent colonoscopy, which did not reveal any signs of polyposis. On subsequent follow up visits the patient is doing well.

## DISCUSSION

Although the cell of origin of desmoid tumors is not clearly defined, it is suggested that desmoid tumors arise from mesenchymal progenitor cells.^4^ they are usually thought to be locally invasive but non-metastasizing.^5^ Most cases occur sporadically, with most cases having mutation in the beta-catenin pathway, in the gene CTNNB1.^6^ They usually arise in the mesentery or retroperitoneum, when intraabdominal.^3^ Desmoid tumors have also been associated with familial adenomatous polyposis (FAP), usually caused by a mutation in the APC gene when abdominal. These arise in the female patient, during or after pregnancy, and usually following abdominal trauma. The most affected age group is 15 to 50 years of age.^3^

One of the most common differential diagnosis is the more commonly encountered gastrointestinal stromal tumor (GIST). The most common sarcoma, or mesenchymal tumors, of the gastrointestinal (GI) tract are gastrointestinal stromal tumors, or commonly referred to as GISTs.^7^ Initially, the pathogenesis was obscure, but recently they have been described as arising from the interstitial cells of Cajal, primarily within the stomach in the muscular layer (50-70%), and small intestine (20-30%).^8^ Most of the cases of GIST have been attributed to mutations in the tyrosine-protein kinase KIT, or cluster of differentiation 117 (CD117), encoding proto-oncogene named c-KIT.^9^ Histologically it is difficult to differentiate the two, as both have characteristic proliferation of spindle cells.^3^ The differentiation is done by use of immunohistochemistry, which usually notes expression of CD1 1 7 marker more weakly in fibromatosis compared to GIST. Further differentiation can be done with the detection of b-catenin and APC mutations in favor of desmoid tumors.

The gold-standard for treatment of fibromatosis is by wide local excision, and reconstruction of the defect.^1,10-11^ Positive margins or incomplete surgical removal can lead to recurrence of the tumor, hence caution is advised during debulking surgery.^1^ Abdominal wall tumors seem to have a better prognosis than the ones presenting at the extremities.^10-11^ Adjuvant therapy comprises of radiation therapy, which has also been applied as a definitive therapy, hormonal or nonsteroidal anti-inflammatory drug (NSAID) therapy (first-line systemic therapy for unresectable, recurrent or progressing desmoid), and anthracycline-based chemotherapy regimen.^11^ Anthracyclines may be combined with methotrexate, vinblastine and cisplatin.^11^

As it is a rare disease, it is vital to consider desmoid tumor as a differential for anterior abdominal wall mass. Thus, it can be difficult to differentiate between intra abdominal fibromatosis and gastrointestinal stromal tumors. The only available means of differentiation at present is the use of immunohistochemistry staining. However, excision with wide surgical margins should form the mainstay of treatment of such tumors, regardless of certainty of diagnosis.
